# Innate immune responses to gut microbiota differ between oceanic and freshwater threespine stickleback populations

**DOI:** 10.1242/dmm.021881

**Published:** 2016-02-01

**Authors:** Kathryn Milligan-Myhre, Clayton M. Small, Erika K. Mittge, Meghna Agarwal, Mark Currey, William A. Cresko, Karen Guillemin

**Affiliations:** 1 Biology Department, Institute of Ecology and Evolution, University of Oregon, Eugene, OR 97403, USA; 2 Biology Department, Institute of Molecular Biology, University of Oregon, Eugene, OR 97403, USA; 3 Biological Sciences, University of Alaska Anchorage, Anchorage, AK 99508, USA

**Keywords:** Neutrophils, Gnotobiotic, Stickleback, Animal model, Germ-free, Fish

## Abstract

Animal hosts must co-exist with beneficial microbes while simultaneously being able to mount rapid, non-specific, innate immune responses to pathogenic microbes. How this balance is achieved is not fully understood, and disruption of this relationship can lead to disease. Excessive inflammatory responses to resident microbes are characteristic of certain gastrointestinal pathologies such as inflammatory bowel disease (IBD). The immune dysregulation of IBD has complex genetic underpinnings that cannot be fully recapitulated with single-gene-knockout models. A deeper understanding of the genetic regulation of innate immune responses to resident microbes requires the ability to measure immune responses in the presence and absence of the microbiota using vertebrate models with complex genetic variation. Here, we describe a new gnotobiotic vertebrate model to explore the natural genetic variation that contributes to differences in innate immune responses to microbiota. Threespine stickleback, *Gasterosteus aculeatus*, has been used to study the developmental genetics of complex traits during the repeated evolution from ancestral oceanic to derived freshwater forms. We established methods to rear germ-free stickleback larvae and gnotobiotic animals monoassociated with single bacterial isolates. We characterized the innate immune response of these fish to resident gut microbes by quantifying the neutrophil cells in conventionally reared monoassociated or germ-free stickleback from both oceanic and freshwater populations grown in a common intermediate salinity environment. We found that oceanic and freshwater fish in the wild and in the laboratory share many intestinal microbial community members. However, oceanic fish mount a strong immune response to residential microbiota, whereas freshwater fish frequently do not. A strong innate immune response was uniformly observed across oceanic families, but this response varied among families of freshwater fish. The gnotobiotic stickleback model that we have developed therefore provides a platform for future studies mapping the natural genetic basis of the variation in immune response to microbes.

## Abstract (precis)

**Summary:** We developed a gnotobiotic threespine stickleback system to identify the cellular and genetic bases for variation in inflammatory responses to microbiota.

## INTRODUCTION

Animals and their associated microbial communities co-exist in dynamic relationships involving complex networks of interactions and signals (McFall-Ngai et al., 2013). These resident microbes provide important services to the host such as nutrient acquisition and pathogen exclusion (Khosravi and Mazmanian, 2013; Tremaroli and Bäckhed, 2012). They also serve a vital role in stimulating the maturation and functioning of the host's immune system (Kamada and Núñez, 2014). Immune cells, including neutrophils, are always present in the gastrointestinal (GI) tracts of healthy individuals, poised to respond to changes in the microbial community. The immune system, while protecting the host from pathogens, must concurrently maintain a homeostatic relationship with, or tolerance of, the beneficial resident microbial community. Loss of this immune tolerance is a characteristic of inflammatory bowel disease (IBD) (Graham and Xavier, 2013). Individuals with IBD suffer from chronically inflamed GI tracts characterized by a higher number of neutrophils (Chassaing and Gewirtz, 2014; Greenblum et al., 2012). Immunosuppression drugs, often used to treat IBD, cause a hypo-inflammatory state that increases susceptibility to pathogen infection (Hansen and Sartor, 2015; Lawley et al., 2008). The normal range of innate immune responses to resident microbiota that occurs within populations of healthy individuals has not been extensively explored.

The inflammatory state of the host is influenced by the host's genetic background and by environmental factors, such as microbial exposures (Huttenhower et al., 2014). Both host genetics and microbial exposures contribute to shaping the composition of the microbiota, which in turn can affect the state of host inflammation (Bolnick et al., 2014c; Goodrich et al., 2014; Knights et al., 2014; Koren et al., 2012). The complexity of the host genetic underpinnings of the microbiota–immune-system relationship is highlighted by the genetic complexity of IBD, in which this microbiota–immune-system relationship is perturbed. At least 163 genetic loci in the human genome have been linked to IBD (Jostins et al., 2012), all of small effect size. Collectively, these genes function in many aspects of immune system signaling and mucosal barrier integrity (Graham and Xavier, 2013), functions that are highly conserved across vertebrate species (Renshaw and Trede, 2012). Vertebrate models are therefore valuable for understanding pathologies of microbiota–immune-system relationships.

To understand the role of microbiota in inflammatory diseases, animal models can be compared in the presence or absence of microbes. Genetic manipulations and chemical treatments have been used to model IBD in mice and zebrafish (Hansen and Sartor, 2015; Oehlers et al., 2013; Sartor, 2009; Xavier and Podolsky, 2007). In almost all cases, these animal models have shown reduced gut inflammation when raised in germ-free conditions, indicating that the increases in neutrophils and other immune cells are due to responses to the microbiota. Most genetic models of IBD have employed null mutations of genes involved in major signaling pathways required for host-microbe interactions (Frantz et al., 2012; Larsson et al., 2012; Vijay-Kumar et al., 2010), or ablation of whole populations of immune cells (Kawamoto et al., 2014). Although these manipulations result in drastic alterations in intestinal inflammation and might represent extreme disease states of the host, they do not reflect the more subtle and complex genetic variation that influences immune interactions with microbes in natural populations, such as human populations at risk for IBD (Jostins et al., 2012). Furthermore, many genes of small effect might collectively have a large impact on IDB development that might often be modulated by the environment. Changing the microbial environment and diet can lead to differences in IBD etiology between genetically variable individuals (Huttenhower et al., 2014). Diseases of microbiota dysbiosis are therefore canonical complex quantitative traits influenced by many genes interacting with one another and the environment.

The current inbred animal models used for IBD research have little of such complex genetic variation and therefore have limited value for identifying diagnostic or predictive genetic markers of complex diseases (DeVoss and Diehl, 2014). We developed the threespine stickleback fish, *Gasterosteus aculeatus*, as an evolutionary mutant animal model (Albertson et al., 2009) to study the inflammatory response to resident intestinal microbiota in a host with natural genetic variation. Stickleback have long been used as a model organism to study the evolution of complex traits such as behavior and ecological morphologies (Bell and Foster, 1994), and have recently become a pre-eminent model for identifying the polygenic basis of natural phenotypic variation (Colosimo et al., 2004, 2005; Cresko et al., 2004; Glazer et al., 2015; Greenwood et al., 2013; Kimmel et al., 2012; Leinonen et al., 2012). Throughout the Northern Hemisphere, oceanic stickleback have invaded freshwater environments and evolved in response to these new conditions. Oceanic and freshwater habitats differ markedly in environmental variables such as salinity, temperature, predation regimes, prey organisms and microbes (Bolnick et al., 2014b; Smith et al., 2015). Freshwater populations of stickleback have often evolved in parallel phenotypically (Cresko et al., 2007) and genetically (Hohenlohe et al., 2010a; Jones et al., 2012). Examination of these populations has allowed researchers to gain insight into the natural genetic variation that affects conserved complex biological processes such as craniofacial and dermal bone development (Kimmel et al., 2005), pigmentation (Jones et al., 2012; Miller et al., 2007) and behavioral phenotypes (Wark et al., 2011), among other complex traits (Albert et al., 2008; McGuigan et al., 2011).

We hypothesized that innate immune responses to microbiota might be an additional complex phenotype that varies between oceanic and freshwater stickleback. Previous studies have shown that the stickleback adaptive immune response has evolved major histocompatibility complex class II (MHCII)-specific responses to eukaryotic parasites (Eizaguirre et al., 2012; Matthews et al., 2010) and microbiota (Bolnick et al., 2014c). Additionally, exposure to parasites in stickleback results in population differentiation in the innate immune response (Lenz et al., 2013). We adapted the threespine stickleback model to examine the innate immune response to intestinal microbiota in two closely related populations of stickleback, an oceanic population similar to the ancestral population and a derived freshwater population; these two populations have likely been exposed to different microbial communities for at least 10,000 years (Hohenlohe et al., 2010a). We find that these two stickleback populations share many gut-associated microbes and have similar intestinal development. We also developed methods to rear stickleback germ-free to investigate the immune response to their microbiota. Interestingly, we find that the inflammatory response to gut microbiota in the ancestral oceanic population is more robust than that of the derived freshwater population, highlighting how even closely related populations can evolve distinct immune responses to microbes. This differential innate immune response was observed both when fish were exposed to the complex microbial communities in the shared, low-salinity laboratory water system, as well as when exposed to a single gut microbiota member common to the two populations. Use of this gnotobiotic stickleback model will allow us to dissect the genetic and microbial factors that modulate host intestinal inflammatory responses and will provide new approaches to studying the genetic variation underlying human diseases of microbiota–immune-system dysregulation.

## RESULTS

### Wild-caught and laboratory-reared stickleback share a core gut microbiota

**Fig. 1.**
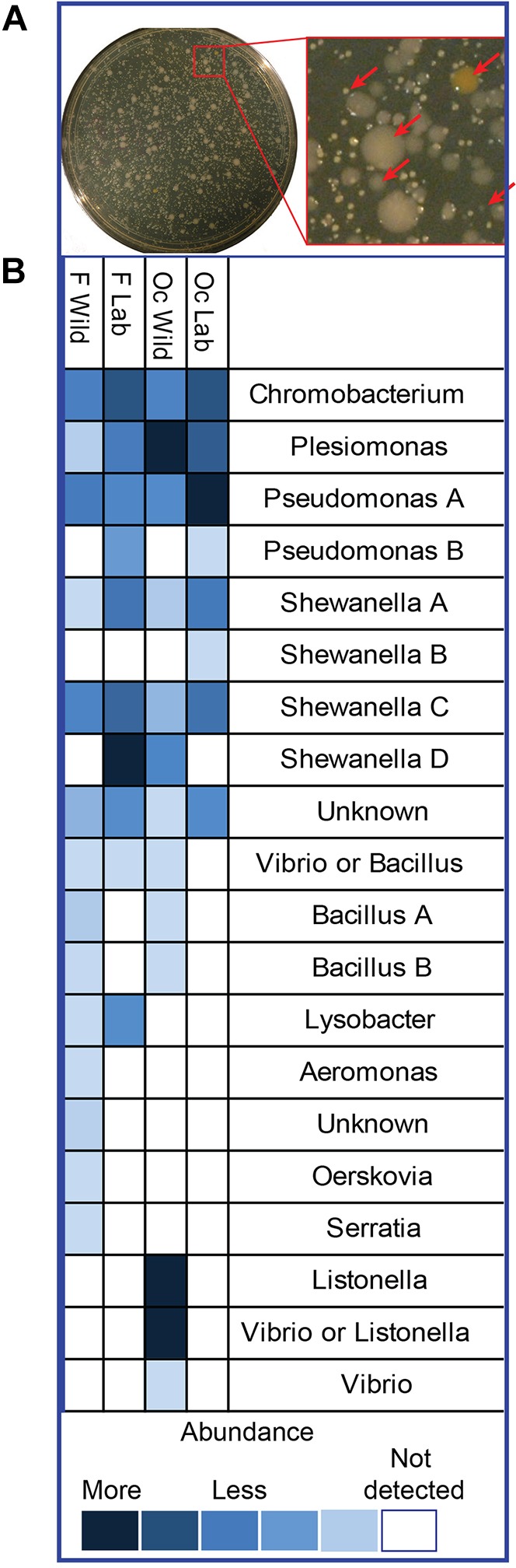
**Bacterial isolates collected from lab-raised and wild-caught stickleback.** Bacteria were isolated from the guts of lab-raised Alaskan adult stickleback and wild-caught Oregon freshwater and oceanic populations. (A) Colony types were based on physical characteristics like color, size and morphology. Representative plate shown. Red arrows point to five different colony types. (B) Relative abundance was estimated based on the number of colonies present in diluted samples. Colonies were identified based on sequencing of the 16S ribosomal RNA gene. Identification to the level of genus is provided. Colony types in the same genus that have different phenotypes or species identification are given letters. Colony types that had 16S sequences that could belong to two different genera are listed as such. Unknown indicates that the colony type could not be identified by 16S sequencing. Dark blue represents the most-abundant type in a population, light blue is the least abundant, and white squares indicate the absence of the colony type in that population. Oc, oceanic; F, freshwater.

To establish whether it would be feasible to compare immune responses to microbiota across stickleback populations, we first characterized the gut microbes from wild-caught and laboratory-reared stickleback from both oceanic and freshwater populations using culture-based assays. The adult populations examined include wild stickleback caught from a coastal oceanic Oregon population, a freshwater river Oregon population, a laboratory-raised population derived from an oceanic Alaskan population (Rabbit Slough), and a laboratory-raised freshwater Alaskan population (Boot Lake) (Table S1). The original wild Alaskan populations evolved separately in two different environments [oceanic in a high-salt-water open ocean with yearly migration to a low-salinity slough, and freshwater in an isolated freshwater lake (Cresko et al., 2004)], but were maintained in the laboratory in identical diet and intermediate salinity conditions. Gut bacteria from each population were analyzed by plating homogenized intestines on non-selective, nutrient-rich agar in aerobic and microaerobic conditions. Colony types based on size, shape, color and other physical attributes were quantified (Fig. 1A) and identified by PCR amplification and sequencing of the 16S ribosomal RNA (rRNA) gene. Although this culture-based survey was not comprehensive, it allowed us to compare the readily cultured subset of the microbiota from each fish population (biased toward fast-growing, non-fastidious microbes) and yielded useful bacterial isolates for further gnotobiotic experimentation.

Given that the oceanic and freshwater populations have evolved in environments with very different salinity, temperatures, predator/prey availability and other environmental factors (Brophy et al., 2011; Cresko et al., 2004; Willacker et al., 2010), we expected the microbiota of the oceanic populations to be different from the freshwater populations. Because the laboratory populations are raised in a controlled environment with presumably very different microbial exposures, we entertained the possibility that the laboratory-raised population would have different microbiota from the wild populations. However, we found that, although the wild-caught fish had several additional microbial colony types compared to the laboratory-reared populations, stickleback populations raised in the laboratory shared many of the bacteria genera with the wild populations. The isolated colony types from all four fish populations made up bacteria from at least 12 different genera, representing the three phyla of Proteobacteria, Firmicutes and Actinobacteria (Fig. 1B). Several microbes that were phenotypically similar to fungi were not identifiable by 16S rRNA sequencing. In correspondence with observations in humans, mice and other fish, stickleback gut microbiota varied between individuals: no one colony type was observed in all fish. Additionally, colony types that were identified as *Pseudomonas* or *Chromobacterium* by 16S rRNA gene sequencing were highly prevalent and abundant in all populations. *Shewanella* and *Plesiomonas* were also found in fish from all four populations. Although not found in every individual, they were in high abundance when present. The finding of common microbes in all populations is striking given that the wild-caught fish were maintained in their native water, which varies in salinity and other chemical and biological components from each other and from the laboratory water.

Only a few colony types were found exclusively in one population. For instance, one colony type identified by 16S rRNA gene sequencing as a *Shewanella* species was found in the intestines of laboratory-raised oceanic adults, but was undetected in the laboratory-raised freshwater adults, despite the shared water system of these two populations. Isolates like these could represent bacterial species that are under specific selection by the host. Stickleback from wild-caught oceanic populations had three microbial colony types that were not found in any other populations; all were identified to be *Vibrio* or the closely related *Listonella*. Freshwater wild stickleback were the only fish with four unique colony types, identified by 16S rRNA gene sequencing as *Oerskovia*, *Serratia*, *Aeromonas* and one unidentifiable; none were highly abundant or found in all freshwater wild fish. Owing to the similarity between the microbiota of the wild and laboratory-raised populations, we concluded that the laboratory-raised stickleback populations were suitable for studying naturally occurring host-microbe interactions. Laboratory-raised Alaskan populations were used for the following studies.

### Conventionally reared oceanic and freshwater stickleback intestines are colonized by 12 days post-fertilization

**Fig. 2.**
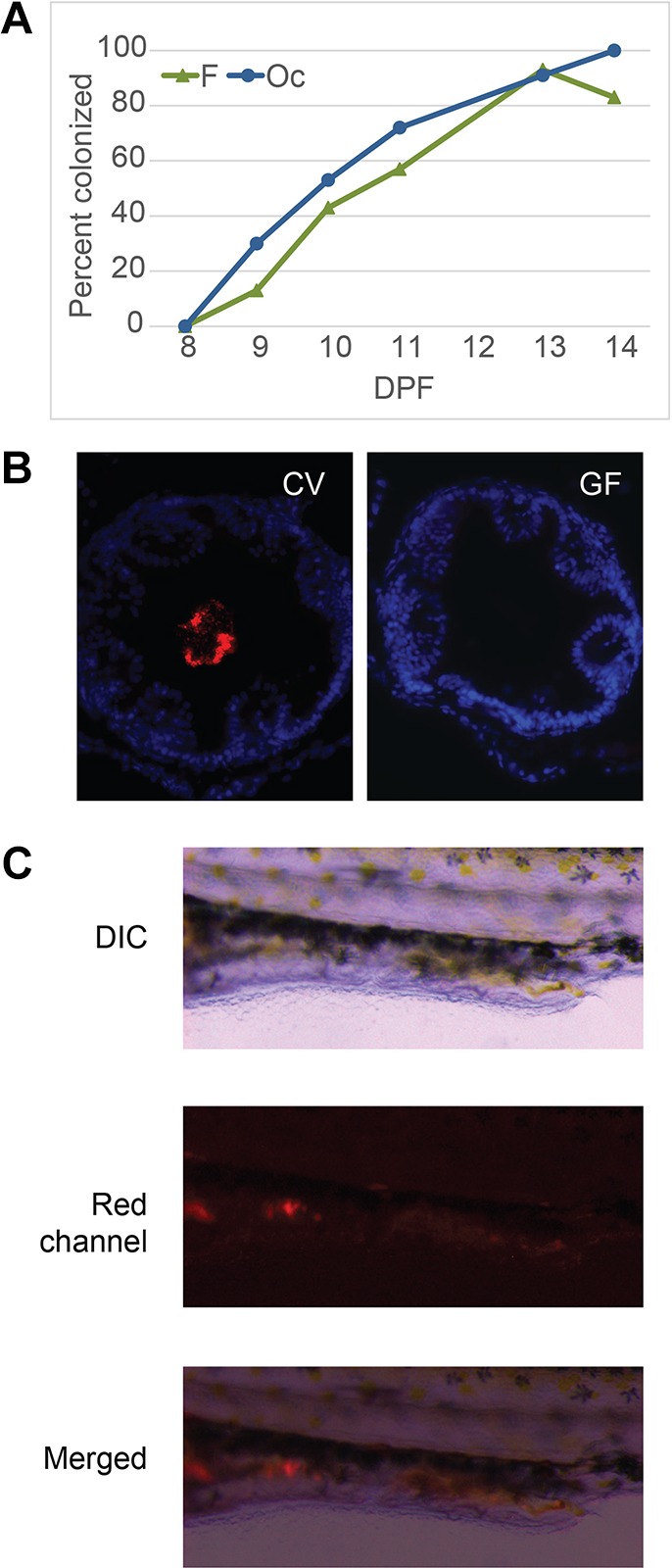
**Gut colonization and gnotobiotic manipulation.** (A) Gut colonization was determined by plating dissected guts daily between 8 and 14 dpf. The average percent of gut colonized from each experiment are reported. Oc, oceanic stickleback (blue line); F, freshwater stickleback (green line). (B) Sterilization of germ-free (GF) stickleback eggs was confirmed with *in situ* hybridization with a pan-bacterial probe (red) and other methods described in the text; conventional (CV) fish shown for comparison. DAPI-stained DNA (blue) indicates epithelial gut cells. (C) Representative 14 dpf live oceanic fish colonized with *Pseudomonas* sp. isolate from a stickleback gut engineered to express red fluorescent protein (KMM0021:RFP). Top panel: differential interference contrast (DIC) image; middle panel: red channel; bottom panel: merged image of the DIC and red channel.

Little is known about the early intestinal development of stickleback, including at which time point microbes first colonize the gut. Therefore, to examine host-microbiota interactions in oceanic and freshwater stickleback, we determined when their intestines were initially colonized. Consistent with previous reports (Swarup, 1958), we observed that stickleback larvae emerge from their chorion between 8 and 9 days post-fertilization (dpf) in both the oceanic and freshwater populations when raised in standard conditions of 20°C (Cresko et al., 2004). As with other fish, larval stickleback initially acquire nutrients by absorbing their endogenous yolk until the intestinal tract is open from the mouth to the vent. In both populations, all visible yolk was absorbed by 14 dpf, suggesting that the intestinal tract should be open and functioning. To determine when stickleback guts are colonized, we performed culture-based analysis of guts dissected daily between 9 dpf and 14 dpf. We found that, although colonization timing varied by experiment, more than half the fish in both populations were colonized by 11 dpf, and nearly all fish in both populations were colonized by 13 dpf (Fig. 2A).

### Developing stickleback as a gnotobiotic model system

In order to examine the impact of the microbiota on stickleback development, we established methods to rear stickleback larvae in the absence of microbes. We adapted the well-established gnotobiotic manipulation of zebrafish (Milligan-Myhre et al., 2011; Pham et al., 2008) for use with stickleback, modifying the protocol to accommodate the relatively slower development and more resistant chorion of stickleback (Fig. S1A). Using these methods, we were able to rear stickleback larvae in the absence of microbiota [germ-free (GF)] or with a single bacterial isolate [monoassociated (MA)] until 14 dpf, when the egg yolk was absorbed and fish required exogenous nutrients. For these studies, we examined the effect of monoassociating stickleback with the most abundant bacterial genera found in all populations, *Pseudomonas*, using isolate KMM0021 from lab-raised adult fish. We compared GF and MA larvae to larvae reared under standard laboratory conditions with complex communities of microbes [conventional (CV)]. Sterility of GF fish was confirmed by PCR amplification of the 16S rRNA gene from water samples and plating of water and gut contents of representative fish from each flask (data not shown), or fluorescent *in situ* hybridization (FISH) with bacteria-specific probes in fixed, cross-sectioned fish (Fig. 2B). FISH also revealed that, at 14 dpf, the lumen of the gut is colonized with a robust population of bacteria, similar to colonization in larval zebrafish (Bates et al., 2006).

The gnotobiotic stickleback system allowed us to examine in more detail the colonization dynamics of the intestine during development. For these experiments we engineered a stickleback gut isolate of *Pseudomonas* strain KMM0021 to express a red fluorescent protein (RFP). This strain, KMM0021:RFP, was introduced into oceanic and freshwater GF fish at 10 dpf, and imaged with a fluorescent dissecting microscope starting at 11 dpf. In agreement with our initial colonization data (Fig. 2A), we observed that fluorescent *Pseudomonas* colonized the full length of the intestines of both oceanic and freshwater fish by 14 dpf (Fig. 2C, oceanic shown), with all fish colonized by 14 dpf (data not shown). This finding further supports our conclusion that intestinal colonization is completed by 14 dpf in both oceanic and freshwater stickleback, and justifies our use of this time point in the following experiments.

We used gnotobiology to examine the role of microbes in stickleback gut development between oceanic and freshwater populations. We employed hematoxylin and eosin (H&E) staining to examine gut morphology of both oceanic and freshwater populations in four separate regions of the digestive system: the esophagus, stomach, and anterior and posterior intestine (Fig. S1B). In stickleback, the esophagus is distinguishable from the stomach by a high density of large, round cells and fewer folds compared to the stomach. The stomach has been described previously (Knight and Burnstock, 1993), and it is separated from the intestine by a contracted junction. The anterior intestine is separated from the posterior intestine by a contracted junction and the posterior gut terminates at the anal vent. To observe overall development over time, fish were collected daily between 9 dpf and 14 dpf. In the majority of fish from both populations, the anterior and posterior intestines had deep folds by 12 dpf, and the junction between the anterior and posterior intestines was fully formed by 14 dpf. We observed no differences in the gross morphology of the intestines of oceanic or freshwater fish raised CV or GF at 12 or 14 dpf (data not shown).

**Fig. 3.**
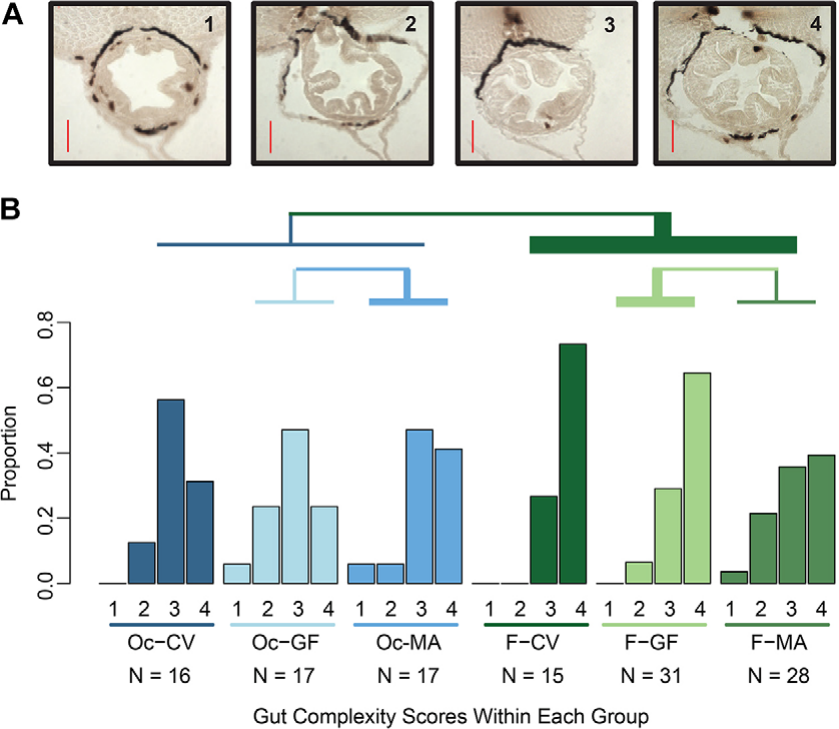
**Stickleback gut development differs between populations but is not generally affected by the presence of microbes.** (A) Scoring of the gut at 14 dpf. Representative images of scored guts are shown. Score 1=little to no intestinal folds; 2=small, undeveloped folds that are unevenly distributed; 3=unevenly distributed folds; 4=fully developed, evenly distributed folds. Scale bars: 25 μm. (B) Graph indicates the proportion of fish in each treatment with gut complexity levels 1 through 4 for conventional (CV), germ-free (GF) or monoassociated with *Pseudomonas* stickleback gut isolate KMM0021 (MA) for oceanic (Oc) and freshwater (F) populations. On average, Oc fish (blue groups) demonstrated lower complexity scores than F fish (green groups) (*P*=0.007). The only significant microbiota-related effect was a statistical interaction with host population in which complexity is higher in Oc-MA relative to Oc-GF fish but lower in F-MA relative to F-GF fish (*P*=0.02). Total numbers of fish scored per group are indicated below the group names. Lines above the figure indicate comparisons with differences in gut development, where the color of the line indicates the groups that were compared, and the width of the line indicates the direction and magnitude in the odds ratio for each comparison.

Examination of the morphology of the intestinal folds in CV fish across larval development revealed that the anterior gut had few folds and was relatively smooth between 8 dpf and 10 dpf, but, by 17 dpf, folds of up to 25 µm were visible within the intestine. To determine whether oceanic or freshwater populations raised in CV or GF conditions differed in fold morphology at 14 dpf, after microbial colonization of the gut, sections of distal intestine were scored for gut fold complexity, following a scoring system that we established from 1 (no folds) to 4 (deep folds) (Fig. 3A). We detected no overall effect of microbiota on gut morphology between CV and GF fish (ordered logistic regression*, t*=0.208, *P*=0.835, Fig. 3B). Host population, however, did generally affect the probability of being assigned a particular gut score (ordered logistic regression, *t=*2.681, *P=*0.007). Specifically, belonging to the freshwater population, as opposed to the oceanic population, increased odds of having a more developed gut by 5.189 times (odds ratio 95% C.I.=1.582-17.743, Fig. 3B). One class of interaction between host population and microbiota also influenced gut score (ordered logistic regression, *t*=−2.326, *P*=0.020). Specifically, belonging to the freshwater population and being monoassociated with the stickleback gut *Pseudomonas* isolate KMM0021 (MA) relative to GF decreased the odds of having a more developed gut by 3.614 times, whereas belonging to the oceanic population and being MA relative to GF increased the odds of having a more developed gut by 2.017 times (Fig. 3B). This host population-by-microbiota interaction was driven by the MA treatment because testing the model with only GF and CV treatments did not yield a significant interaction term (ordered logistic regression, *t*=0.593, *P*=0.553). These data indicate that colonization with this particular *Pseudomonas* species stimulates gut development in a population-specific manner, highlighting how genetically distinct hosts might show differential responses to individual microbes.

### Genetically distinct populations of oceanic and freshwater stickleback differ in their intestinal neutrophil response to microbiota

**Fig. 4.**
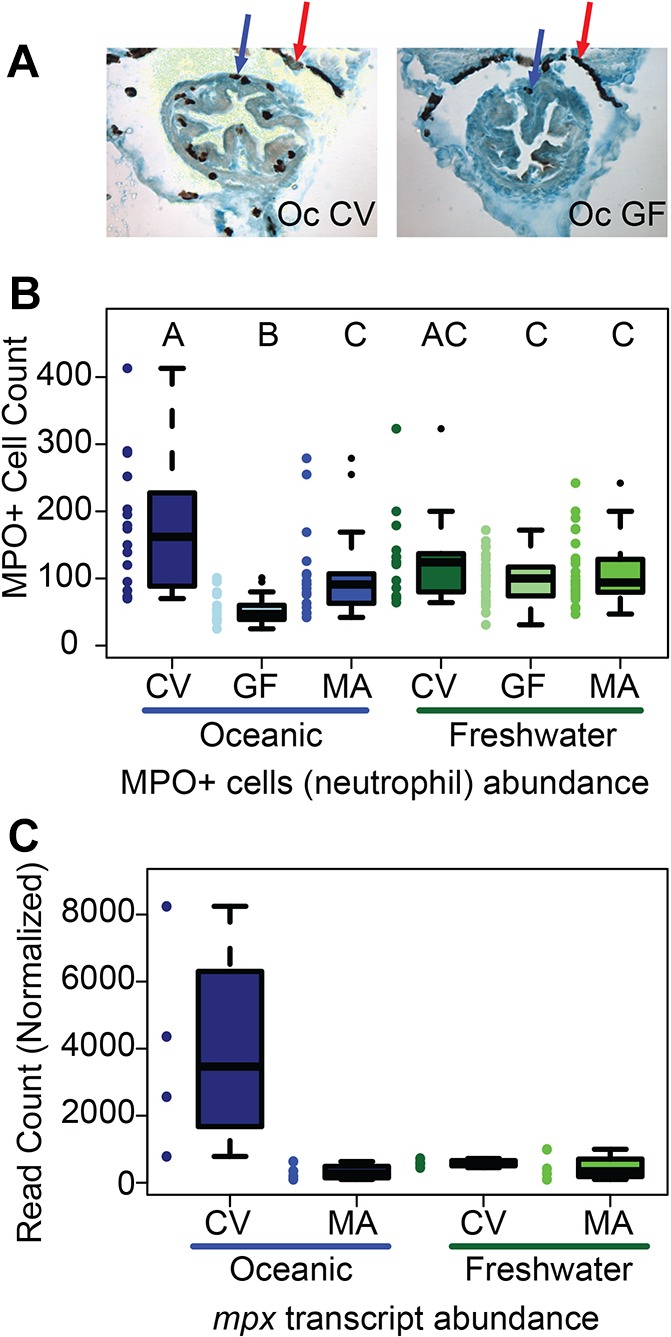
**Oceanic populations mount a robust innate immune response to microbiota, whereas freshwater populations do not.** (A) To examine the innate immune response to microbiota, myeloperoxidase-positive neutrophils (MPO+) were stained and counted. MPO+ cells are brown. Red arrows indicate black pigment at the periphery of the intestine; blue arrows indicate representative MPO+ cells within the intestinal epithelium. Oc, oceanic; CV, conventional; GF, germ-free. (B) The number of MPO+ cells per gut were counted per fish. Colored dots represent the number of MPO+ cells in individual fish, and boxplots represent distributions within conventional (CV), germ-free (GF) and *Pseudomonas* isolate KMM0021 monoassociation (MA) treatments. In Oceanic fish, the number of MPO+ cells differs across the three microbe treatments, as indicated by the non-overlapping letter groupings ‘A’, ‘B’ and ‘C’. In freshwater fish, all three microbe treatments are equivalent regarding MPO+ cell number (all three letter groupings overlap). This population-specific microbial effect on neutrophil abundance is reflected by a statistical interaction (*P*<0.0001). See text for details. (C) Neutrophil marker *mpx* transcript levels from an RNA-seq experiment involving CV and MA fish demonstrate the same population-by-microbiota interaction (*P*=0.005). Colored dots represent *mpx* expression levels in guts of individual fish (*n*=4 fish per population-treatment combination), and boxplots show within-group distributions.

Gut microbiota are known to modulate host inflammation (Bates et al., 2007; Galindo-Villegas et al., 2012; Kanther et al., 2014; Maslowski et al., 2009; Rawls et al., 2004). Therefore, we examined the innate immune responses to microbiota colonization in the oceanic and freshwater populations. For these experiments, oceanic and freshwater families were divided into three flasks and were raised CV, GF or mono-associated with the *Pseudomonas* stickleback gut isolate KMM0021 (MA). Intestinal neutrophils were identified based on myeloid peroxidase (MPO) enzyme activity, which is a well-characterized neutrophil biomarker in zebrafish, mouse and humans (Kolaczkowska and Kubes, 2013; Renshaw et al., 2006) (Fig. 4A). MPO-positive cell numbers were quantified along the length of the intestine in histological sections from both oceanic and freshwater populations for each of the three treatment groups. We detected no overall effect of host population on neutrophil count (ANOVA, *F*=0.636, *P*=0.483), but a significant overall effect of microbiota (ANOVA, *F*=16.267, *P*<0.0001) (Fig. 4B). This result indicates that hosts mount an immune response to the microbiota as measured by the increased presence of neutrophils.

In our analysis of the data in Fig. 4B and other experiments, we found that, in addition to the effect of microbiota on neutrophil presence, we also detected a significant interaction between host population and microbiota with respect to the quantity of neutrophils (ANOVA, *F*=7.664, *P*<0.0001) (Fig. 4B), meaning that the response to the microbiota depends on the host population. Consistent with this interaction, we identified three different population-treatment combination groups with respect to neutrophil count, based on post hoc pairwise comparisons: a high-inflammation group composed of CV fish (‘A’), a low-inflammation group comprising GF oceanic fish (‘B’), and an intermediate group composed of MA oceanic fish and all freshwater fish (‘C’) (Fig. 4B).

In order to verify that these differences were not explained by intrinsic size differences between populations, we measured the standard length of oceanic and freshwater fish. Freshwater fish were, on average, longer than oceanic fish at 14 dpf (mean lengths: oceanic=6.50±0.062 mm; freshwater=7.27±0.046 mm; *F*=108.39, *P*<0.001), and GF fish were marginally longer than CV fish (mean lengths: GF=7.10±0.064 mm; CV=6.92±0.050 mm; *F*=6.38, *P*=0.012). An interaction between population and microbiota also influences standard length (*F*=18.26, *P*<0.001), but it is insufficient to explain the population difference in innate immune response, as the magnitude and direction of this interaction are fundamentally different from that observed for neutrophil count (Fig. S1C; Fig. 4B). We also compared the length of fish to the number of neutrophils present in the gut and observed no relationship between the number of neutrophils found in the gut and the length of the fish (*r*^2^=0.01929). Differences in the overall length of the fish were not enough to explain the differences observed in the neutrophil numbers in the populations. Additionally, we examined whether differences in neutrophil numbers could be explained by the overall developmental maturity of the intestine. Importantly, we detected no overall effect of MPO-positive cell count on the probability of being assigned a particular gut morphology score (ordered logistic regression, *t*=1.193, *P*=0.233, Fig. S2).

To determine whether colonization with microbiota affected all intestinal cell types in a population-specific manner, we also examined the number of intestinal goblet cells, which produce mucus. Intestinal mucus is known to protect the host from pathogenic bacteria by serving as a barrier between the host and microbes, to protect the host from inflammation, and to affect the composition of microbiota (Bel et al., 2014). There are typically fewer mucus-producing cells in the intestines of GF mice (Deplancke and Gaskins, 2001) and zebrafish (Bates et al., 2006) compared to CV animals. To test whether oceanic and freshwater populations regulate mucus-producing cells differently owing to interactions with microbiota, we quantified mucus-secreting cells stained with Alcian blue in stickleback raised CV or GF. We detected no overall effect of host population (ANOVA, *F*=2.939, *P*=0.229), microbiota (ANOVA, *F*=0.884, *P*=0.349) or population-by-microbiota interaction (ANOVA, *F*=1.5494, *P*=0.2162) on Alcian-blue-positive cell numbers. These data indicate that there is no difference between populations of Alcian blue cell presence at 14 dpf, and gut microbiota do not affect intestinal Alcian blue cell populations (Fig. S1D,E).

Taken together, these results provide evidence that many aspects of development are shared between the oceanic and freshwater populations and occur independently of the microbiota, but the immune response to the microbiota, as measured by neutrophil count, is contingent on the host genetic background.

### Candidate gene analysis confirms neutrophil differences

Next, we performed intestinal transcriptome profiling, using RNA sequencing (RNA-seq), on oceanic and freshwater fish raised in CV conditions or in the presence of *Pseudomonas* KMM0021. We chose to compare the transcriptomes of fish raised in these conditions due to the striking differences in the number of intestinal neutrophils observed between the treatment groups in oceanic and freshwater populations. In concordance with the neutrophil staining results, normalized RNA-seq read counts of the *mpx* gene, a stickleback ortholog encoding the MPO enzyme that we used as our neutrophil marker, revealed an interaction between population and microbiota similar to that observed for the neutrophil count data (Fig. 4C). In oceanic fish, *mpx* read counts were on average 12.578-fold higher in the presence of a complex microbial community, as compared to the presence of *Pseudomonas* alone. In freshwater fish, we observed no significant difference (fold change=1.309) in levels of *mpx* transcript in fish raised in CV versus monoassociation (MA) with *Pseudomonas* (Fig. 4C). False-discovery-rate-adjusted *P*-values for likelihood ratio tests of the effects of population, microbiota and population-by-microbiota interaction were 0.082, 0.027 and 0.005, respectively. Other peroxidase paralogs in the stickleback genome could, in principle, contribute to the observed histological data, but the *mpx* gene expression data are consistent with counts of intestinal neutrophils in the different populations and highlight the power of the RNA-seq method for future discovery of transcriptional inflammatory responses in this model.

### Freshwater populations show variation in the neutrophil response

We chose stickleback as a model system because of the natural genetic variation that exists among populations and also among individuals within a population. We wanted to determine whether the phenotype of the immune response to microbiota varied across families within the same population. Therefore, we quantified the intestinal neutrophils at 14 dpf from offspring of ten different oceanic families and seven different freshwater families. Parents were raised in separate tanks and represent at least three different generations of fish, sampled over 3 years. Although the water system is shared between tanks, microbial composition likely varies over time and between tanks (Burns et al., 2015), ensuring that the phenotypes we observed were not due to one microbial community composition but across several microbial community structures. All animals were rendered GF, and then CV animals were colonized with microbiota from the parental tank to ensure the variation in intestinal inflammation is not due to distinct maternal microbiota coating the chorions in each clutch. Families are defined as the offspring from an individual male and an individual female within a population.

**Fig. 5.**
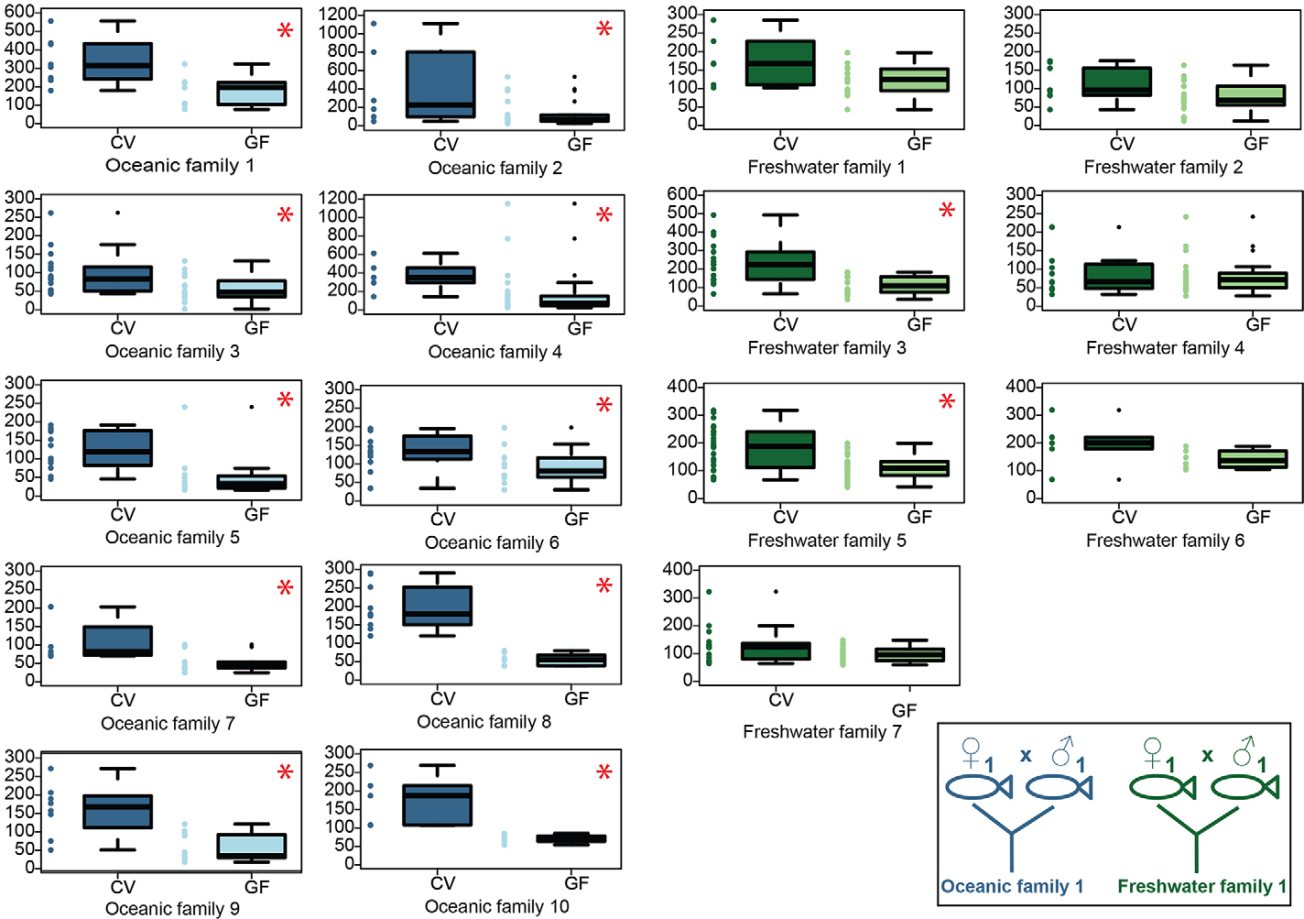
**Neutrophil differences in response to microbiota presence is stable between oceanic families, but variable between freshwater families.** Ten oceanic and seven freshwater families were raised in the presence (CV) or absence (GF) of microbes. The number of MPO+ cells per fish was counted. Individual counts are represented by dots; bars on boxplots indicate median value, boxes represent first through third quartiles, and vertical lines represent outlier fences. For all oceanic families, a difference in MPO+ cells is observed in CV versus GF fish. In five of the seven freshwater families there is no difference in the number of MPO+ cells in CV versus GF fish, but in two families there is a difference. Red asterisks denote families in which the *P*-value is less than 0.05 for the number of MPO+ cells between CV and GF treatments. Families examined in the same experiment are denoted by the same number (i.e. Oceanic family 1 and Freshwater family 1). Diagram in the lower left corner depicts the crossing of a single male and a single female from the oceanic or freshwater population that resulted in a single family.

In the oceanic population, we consistently observed more neutrophils in CV fish compared to GF fish across families. In five of seven freshwater families, we observed no difference in the number of neutrophils in CV fish compared to fish raised GF. In two freshwater families, however, there was considerable difference in the number of neutrophils in CV fish and GF fish (Table 1 and Fig. 5). This variation reflects the genetic heterogeneity that can often be observed within a population. Furthermore, it mirrors the previous observations of increased phenotypic variation in other traits in natural populations of freshwater stickleback (Bell, 2001; Hohenlohe et al., 2010b; Svanbäck and Schluter, 2012; Walker, 1997).

**Table 1.**
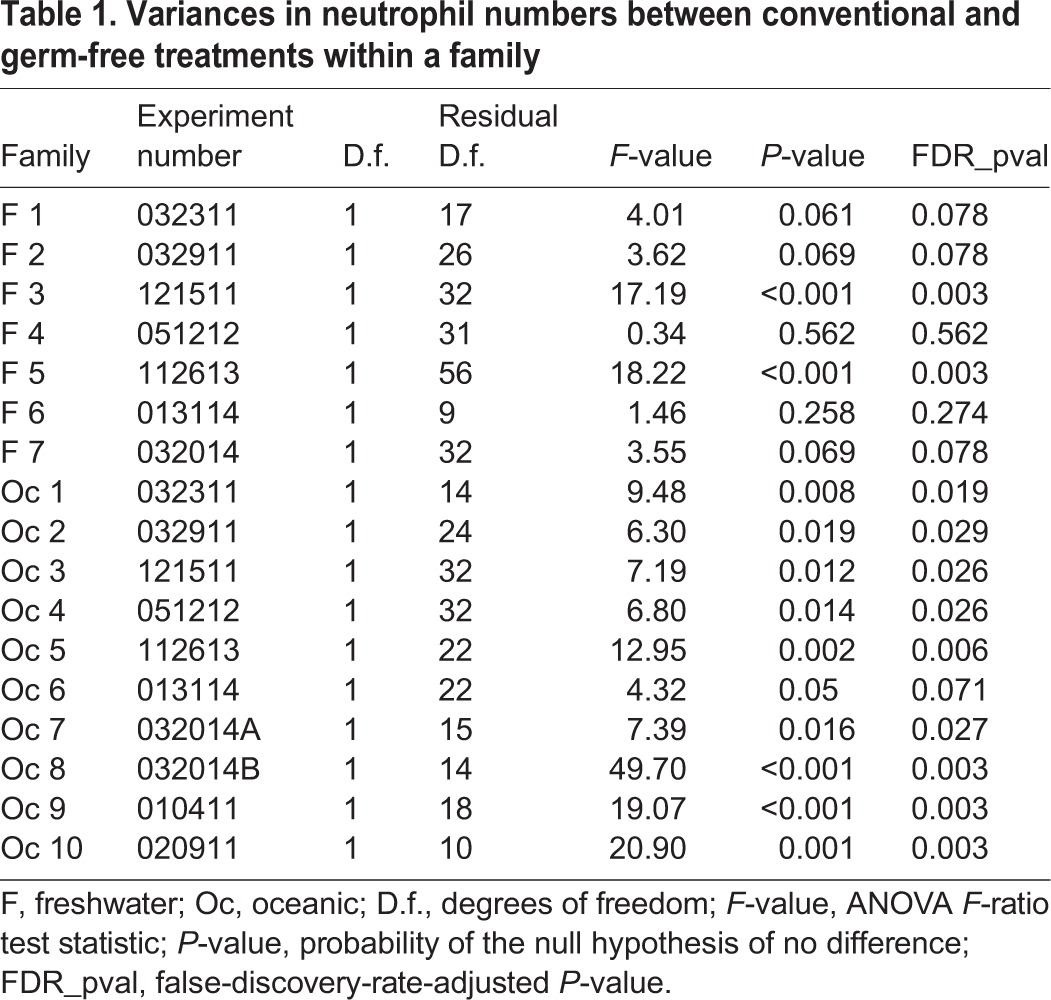
**Variances in neutrophil numbers between conventional and germ-free treatments within a family**

## DISCUSSION

### Gnotobiotic stickleback reveal extensive natural variation in innate immune responses to microbiota

Fish possess robust innate immune systems (Lewis et al., 2014) that have been shown in stickleback to play major roles in protection against pathogens (Kalbe and Kurtz, 2006; Lenz et al., 2013). We hypothesized that, as ocean stickleback populations invaded freshwater habitats, their innate immune system would have adapted to new microbial environments. Therefore, we developed gnotobiotic stickleback as an experimental model system to explore natural variation in host immune responses to gut microbiota, with the long-term goal of identifying genes and pathways that might contribute to human polygenic diseases of excessive inflammatory responses to resident microbes, such as IBD (Xavier and Podolsky, 2007). We focused our analyses on innate immune responses in the larval stickleback intestine because gnotobiotic stickleback can be maintained in sterile conditions until their egg yolk has been absorbed, a period during which the innate immune system can be studied in the absence of the adaptive immune response. In particular, we analyzed the change in the number of neutrophils infiltrating into the gut in response to the presence of the microbiota; neutrophils are an immune cell population that we have previously shown to be responsive to microbiota in zebrafish (Bates et al., 2007).

To measure the innate immune response to microbiota, we developed methods to rear stickleback larvae from embryogenesis through larval stages in the presence or absence of microbes. We found that overall development of the larval gut, based on histological analysis, did not differ between GF- and CV-reared larvae from two evolutionarily distinct oceanic and freshwater Alaskan stickleback populations. Additionally, the overall growth rate, larval gut development and timing of gut colonization were similar between the two populations.

When we compared the quantity of neutrophils between GF- and CV-reared larvae, we observed that the oceanic population had a strong and consistently high immune response to resident microbiota, whereas the response of the freshwater population was less robust and more variable. Strong support for distinct innate immune responsiveness between the two populations came from our gnotobiotic studies. We showed that a conventional microbiota resulted in an increased number of intestinal neutrophils and elevated levels of the neutrophil-specific transcript *mpx* in the oceanic but not the freshwater population.

The differential immune response to microbiota that we observed in the oceanic and freshwater fish could be due to differences in the gut microbiota between the two populations. However, our data argue against this. Our culture-based analysis revealed many shared gut microbes between the oceanic and freshwater populations of fish used in this study, which were maintained on a common, intermediate salinity water system. In addition, the laboratory-reared fish harbored very similar bacterial taxa to those that we cultured from wild-caught oceanic and freshwater stickleback. Many of the bacterial genera that we isolated in culture were also identified in several recent culture-independent surveys of the gut microbiota of stickleback from lake, stream and estuary populations (Bolnick et al., 2014a; Smith et al., 2015).

### Natural variation in innate immune responsiveness likely contributes to microbiota composition and host risk for infections or inflammatory disorders

Our study establishes that host innate immune responsiveness to microbiota is a phenotypically variable trait in natural populations. Strong innate immune responsiveness is likely to confer better protection against pathogen infection, but at the cost of increased risk of excessive inflammation that can be harmful to the host (Miller et al., 2005). Freshwater populations experience relatively constant environmental conditions and microbial communities, whereas the oceanic populations experience a wide variety of salinities, predators and microbial exposures as they migrate from high-salinity oceanic waters to lower-salinity brackish waters to mate (Hahn, 2006; Logares et al., 2009; Smith et al., 2015; Wang et al., 2012; Zwart et al., 2002). The more constant microbial environmental conditions experienced by the freshwater population might have relaxed the selective pressure to maintain strong innate immune responses or conferred a selective advantage for mechanisms that actively dampen innate immune responses. Alternatively, the immune response to microbes in the oceanic population might be elevated due to increased exposure to pathogenic microbes. For example, genetically distinct populations of stickleback from lake and river habitats that vary in the number of parasites present also vary in their immune response to parasites (Lenz et al., 2013; Matthews et al., 2010; Scharsack et al., 2007). Future experiments will explore whether the muted innate immune response that we observed in the Boot Lake population is shared across other freshwater populations, similar to other freshwater phenotypes such as loss of skeletal elements (Barrett et al., 2008; Kimmel et al., 2012).

Our finding of phenotypic variation in innate immune response to microbiota between and within stickleback populations offers the possibility for future studies to map host genetic traits that modulate both innate immune responses and microbiota composition. Mapping of microbiota composition in human twin pairs has revealed variation in the strength of genetic selection on different microbial taxa (Goodrich et al., 2014), which could correlate with their capacity to elicit innate immune responses. Analyses of microbiota from interbred mice have identified several large chromosomal regions associated with microbiota composition (Benson et al., 2010; Leamy et al., 2014; McKnite et al., 2012). These authors did not find any correlation between sequence variation in the major intestinal mucosal immunoglobulin IgA and microbiota composition (Leamy et al., 2014). They concluded that genetic variation in adaptive immunity is not a major contributor to microbiota composition, leaving open the possibility that genetic variation in innate immunity plays a stronger role in shaping the microbiota. A related mapping experiment in fruit flies, which examined microbiota-modulated host genes influencing nutrient acquisition, did not uncover any immune genes (Dobson et al., 2015), possibly owing to the nutritional focus of the study.

We hypothesize that the strong intestinal innate immune responses of fish from the oceanic population would result in a less diverse microbiota compared to fish from the freshwater population, whose dampened immune responses would be more permissive of colonization by diverse microbes. Our culture-based survey of microbiota in wild-caught and laboratory-reared fish was too limited to draw conclusions about microbiota diversity; however, a recent survey of intestinal microbiota from estuary, stream and lake stickleback observed highest alpha diversity in lake-derived populations (Smith et al., 2015), consistent with our expectation. An analogous relationship has been documented in a stickleback population in which low microbiota diversity correlated with high *MHCII* gene heterozygosity and presumably more robust adaptive immunity (Bolnick et al., 2014c). Our expectation of lower microbiota diversity in the presence of a more robust innate immune response is consistent with observations in individuals with IBD, who exhibit a high state of intestinal inflammation and a low microbiota diversity (Conte et al., 2006; Huttenhower et al., 2014).

We speculate that similar genetic pathways that modulate the innate immune responsiveness to microbiota in stickleback also modulate intestinal inflammation in humans and will influence susceptibility to diseases of excessive inflammation, such as IBD. Variability in the immune response to microbiota within and between the freshwater populations and families will aid in mapping genes involved in modulating the intestinal innate immune response to microbiota and will inform future studies into the genetic basis of inflammatory disorders in humans.

## MATERIALS AND METHODS

### Isolation of bacteria from adult sticklebacks

#### Population description

Wild adult Oregon stickleback were collected in the summer of 2010. Wild Oregon freshwater fish were collected from the River Bend site on the McKenzie river (44°4.666′N, 123°1.6′W); adult wild Oregon oceanic fish were collected from Millport Slough in Siletz Bay [44°53′ 14.68″N, 123°59′46.20W, see http://ir.library.oregonstate.edu/xmlui/handle/1957/25763 (Brophy et al., 2011) for annual fluctuations in temperature, salinity, waters levels and other parameters]. Fish were collected in their native water, transported to Eugene, Oregon, and euthanized with a tricaine methane sulphonate solution (MS222).

Alaskan laboratory-reared fish originated from oceanic (Rabbit Slough) or freshwater (Boot Lake) habitats in Southcentral Alaska (Cresko et al., 2004; Hohenlohe et al., 2010a; McGuigan et al., 2010), but have been maintained in the laboratory for 8-11 generations and are now considered ‘lab-raised’ populations. Natural genetic variation in the lab-raised populations is maintained by periodically fertilizing lab-raised eggs with macerated testes from wild fish caught in the same location. Both populations were maintained in separate tanks with a shared 3-4 parts per thousand salinity water-source, mimicking a slightly brackish freshwater environment. Tanks were maintained on a shared recirculating system under identical water conditions, diets, light exposure and temperatures, ensuring that the water and microbial communities within the tanks were shared. Full husbandry protocols are available (http://stickleback.uoregon.edu/index.php/). All work conformed to protocols approved by the University of Oregon Institutional Animal Care and Use Committee requirements.

#### Bacterial isolation

Ten adult sexually mature fish from each wild population and 15 adult sexually mature fish – between 1.5 and 2 years post-fertilization – from each lab-raised population were euthanized with MS222. Intestines were removed aseptically and transferred to 1.8 ml tubes containing sterile stickleback embryo medium (SBEM; 4 ppt Instant Ocean, pH 7.5). Guts were homogenized with sterile pestles and brought up to 1 ml with sterile SBEM. 900 µl of the homogenate was stored at −80°C for future use. The remaining 100 µl of the homogenate was diluted in sterile SBEM and plated on non-selective, nutrient-rich agar plates (tryptic soy agar, lysogeny broth or marine agar). Plates were incubated at room temperature for 2 days either in the presence of oxygen or in microaerobic containers. Individual colony types were identified based on size, shape, color and other physical attributes, quantified, and examples of each colony type were suspended in glycerol and stored at −80°C. When possible, bacteria were identified by the 16S ribosomal RNA gene sequence using universal primers 27F and 1492R (Lane et al., 1985). To determine gut colonization, guts were plated as above. Guts with more than 100 colony-forming units (CFU) per gut (the lowest amount detectable by the assay) were considered colonized. Results in Fig. 1B are combined results from several experiments.

### Crosses and gnotobiology

For each experiment in which juvenile fish were examined (up to 14 dpf), clutches from one to two random lab-raised stickleback females were fertilized with the macerated testes of a single male. Eggs and macerated testes were incubated at room temperature for 2-3 h in 45 mm Petri dishes in SBEM with ampicillin (100 µg/ml), kanamycin (5 µg/ml) and amphotericin (250 ng/ml). Viable, fertilized eggs were disassociated, transferred to 100 mm diameter Petri dishes, washed with SBEM, and incubated for 1-2 h at room temperature. Viable eggs were cleaned with 0.2% polyvinylpyrrolidone-iodine (PVP-I; diluted in SBEM and filter-sterilized) for 10 min, rinsed three times with sterile SBEM, soaked in 0.003% bleach for 10 min, rinsed an additional three times with sterile SBEM, and 20 GF eggs were transferred to 50 ml of sterile SBEM in sterile polystyrene flasks with filter caps (250 or 500 cm^2^; TPP Techno Plastic Products AG, Trasadingen, Switzerland). Flasks containing GF fish remained sealed for the duration of the experiment. Fish were incubated at 20°C until collected at 14 dpf (all MPO studies and Alcian blue quantifications) or earlier in the case of staining for H&E and Alcian blue whole-fish staining. Sterility of the embryos and water was assessed by direct visualization using phase optics at 40× magnification, by culturing media aerobically on tryptic soy agar (TSA) plates at room temperature (approximately 23°C) for 48 h, and by PCR of the bacteria-specific 16S ribosomal RNA gene, as described by Bates et al. (2006).

CV fish were treated the same as GF fish, except that 0.5 to 2 ml of water from tanks containing CV-raised, untreated adult fish was distributed across conventional flasks immediately after cleaning. Although the volume of adult tank water changed between experiments as we experimented with the amount of conventional water to use, all CV fish were treated the with same microbial community within a single experiment. Dead fish were removed to prevent overgrowth of microbes. MA fish were treated the same as GF fish, except that, at 10 dpf, 100 µl of water was removed to check for sterility, dead fish were removed, and either KMM0021 or KMM0021:RFP diluted in sterile SBEM were added to the water to a final concentration of 1×10^5^ CFU/ml at 10 dpf.

### Histology

Unless otherwise stated, fish were euthanized with MS222, fixed with 4% paraformaldehyde overnight at room temperature, embedded in paraffin, cut into 7-µm-thick sections, and mounted on glass slides.

#### Fluorescent *in situ* hybridization of bacterial cells

Tissue sections were deparaffined then probed with a mixture of Eub338-1, Eub338-II and Eub338-III, as previously described for zebrafish (Bates et al., 2006). Sections were covered with VECTASHIELD^®^ HardSet™ Mounting Medium with DAPI (Vector Laboratories, Burlingame, CA) to inhibit photobleaching of the fluorochrome and to stain for DNA, and protected with a coverslip. Fluorescence was observed on a Nikon fluorescent microscope at 60× magnification. Images were captured with a Nikon camera, with the same exposure setting for all images. The images were merged using Adobe Photoshop.

#### H&E staining

Euthanized fish were fixed with either 4% paraformaldehyde as above, or with Boulin's fix overnight, parafinned, sectioned in 7-µm-thick sagittal sections, mounted onto glass slides, deparaffined, stained with H&E following routine staining protocols, and coverslipped.

#### Staining of intestinal neutrophils and goblet cells

Prior to sectioning, fish were stained with p-phenylenediamine and catechol to detect MPO in neutrophils (Sigma, 390A-1KT, St Louis, MO, USA), as per the manufacturer's instructions. Briefly, euthanized fish were rinsed three times in phosphate buffered saline (PBS) with 1% Tween-20, incubated with indicator dye in Trizmal for 30 min at 37°C, rinsed with PBS two times, and fixed again in 4% paraformaldehyde overnight at room temperature. Fish were then mounted in paraffin, sectioned, and either covered with coverslips, or the paraffin was removed and the sections were stained with 0.1% Alcian blue to visualize goblet cells, as previously described for zebrafish (Bates et al., 2006). Alcian-blue- or MPO-positive cells in the posterior gut were counted from the start of the anal vent to the junction between the gut and the stomach on a Leica (Wetzlar, Germany) DM 750 LED microscope. Owing to the large size of stickleback neutrophils, which spanned two sections in many cases, every other section was counted. For fish that did not have well-developed junctions, 150 sections anterior to the vent were counted because this approximated the number of sections from the vent to the stomach in other fish. Scoring of gut development was performed with cross-sectioned fish stained for myeloperoxidase and goblet cells.

The intestinal morphology in MPO- or Alcian-blue-positive sections at either 10 sections (70 µm) or 15 sections (105 µm) anterior from the vent was scored based on evenness and number of folds, with a gut score of 1 indicating no folds, 4 indicating a gut with regularly spaced large folds, and scores of 2 and 3 for gut development between those two extremes.

### Cloning of fluorescently labeled *Pseudomonas* strain

KMM0021 was isolated from lab-raised adult stickleback guts. The strain was fluorescently labeled using a tri-parental cloning method to introduce Gm:tag-dTomato (Singer et al., 2010), via the donor *E. coli* strain TN7T:RFP and helper *E. coli* strain TNS2 (Choi et al., 2005), producing the strain KMM0021:RFP. Fluorescence was confirmed by fluorescent microscopy prior to inoculations, in colonized larval intestines and post-recovery from the intestine of fish.

### Intestinal transcriptome analysis

We surface-sterilized embryos from one freshwater and one oceanic cross and assigned 20 individuals from each clutch to *Pseudomonas* monoassociation (MA) or conventional (CV) microbiota treatments, as described above. At 14 dpf, we euthanized the larvae using MS222, dissected the GI tract from the stomach to the urogenital opening, and isolated total RNA using TRIzol reagent (Invitrogen, Carlsbad, CA), according to the protocol described by Leung and Dowling (2005). We used 200 ng of total RNA from each of four stickleback guts per family-treatment combination (16 total) to generate RNA-seq libraries with the TruSeq mRNA v2 Kit (Illumina, San Diego, CA). Ten million 100-nucleotide (100-nt) Illumina HiSeq2500 reads from each sample were aligned to the Ensembl v75 threespine stickleback reference genome; we counted the number of reads mapped uniquely to each gene model, and we performed normalization and differential expression analysis using the negative binomial generalized linear models implemented in the R statistical package edgeR (Robinson et al., 2010). The RNA-seq data are part of a larger gene-expression study and will be described fully in a future publication.

### Statistical analyses

We carried out all statistical analyses using the R statistical language version 3.0.2 (R Core Team, 2013). To test the effects of host population and microbiota on the inflammatory response we compared the number of MPO-positive cells in the entire gut among individuals from three freshwater and two oceanic families. Individuals in families were randomly split among CV, MA or GF treatments, so we analyzed the data in an unbalanced, partly nested analysis of variance (ANOVA) framework with family as a random blocking factor. This design allowed us to test for fixed effects of population, microbiota and population-by-microbiota interaction on MPO-positive cell number. To better satisfy distributional and mean-variance relationship assumptions of ANOVA, we analyzed square-root-transformed values of MPO-positive cell count.

To ensure that the patterns of inflammatory response related to host population and microbiota were not simply a consequence of corresponding differences in gut complexity among fish, we also compared gut complexity scores on a scale from 1 to 4, as described above. To test the potential effects of host population, microbiota and MPO-positive cell number on gut morphology, we compared the complexity score among the same individuals from the three freshwater and two oceanic families described above using ordinal logistic regression. To simplify interpretation, we excluded family as a blocking factor in the ordinal logistic regression model, pooling individuals from different families but the same population. This design allowed us to test for effects of MPO-positive cell count, population, microbiota and population-by-microbiota interactions on the probability of a fish having a more complex versus less complex gut morphology. We calculated *P*-values for hypothesis tests by comparing the *t-*test statistic values from the ordinal logistic regression against the standard normal distribution. In addition to tests of statistical significance for each term in the model, we calculated 95% confidence intervals (C.I.) for odds ratios.
